# Biomarker Discovery in Liver Disease Using Untargeted Metabolomics in Plasma and Saliva

**DOI:** 10.3390/ijms251810144

**Published:** 2024-09-21

**Authors:** Noah J. Daniels, Courtney E. Hershberger, Matthew Kerosky, Chase J. Wehrle, Roma Raj, Nihal Aykun, Daniela S. Allende, Federico N. Aucejo, Daniel M. Rotroff

**Affiliations:** 1 Department of Quantitative Health Sciences, Lerner Research Institute, Cleveland Clinic, Cleveland, OH 44106, USA; danieln2@ccf.org (N.J.D.); hershbc@ccf.org (C.E.H.); 2 Center for Quantitative Metabolic Research, Cleveland Clinic, Cleveland, OH 44106, USA; 3 Department of HPB Surgery and Liver Transplantation, Cleveland Clinic, Cleveland, OH 44106, USA; 4 Department of Pathology, Cleveland Clinic, Cleveland, OH 44106, USA; 5 Endocrinology and Metabolism Institute, Cleveland Clinic, Cleveland, OH 44106, USA

**Keywords:** hepatocellular carcinoma, NAFLD, liver cirrhosis, liver disease, biomarker discovery, metabolomics

## Abstract

Chronic liver diseases, including non-alcoholic fatty liver disease (NAFLD), cirrhosis, and hepatocellular carcinoma (HCC), continue to be a global health burden with a rise in incidence and mortality, necessitating a need for the discovery of novel biomarkers for HCC detection. This study aimed to identify novel non-invasive biomarkers for these different liver disease states. We performed untargeted metabolomics in plasma (Healthy = 9, NAFLD = 14, Cirrhosis = 10, HCC = 34) and saliva samples (Healthy = 9, NAFLD = 14, Cirrhosis = 10, HCC = 22) to test for significant metabolite associations with each disease state. Additionally, we identified enriched biochemical pathways and analyzed correlations of metabolites between, and within, the two biofluids. We identified two salivary metabolites and 28 plasma metabolites significantly associated with at least one liver disease state. No metabolites were significantly correlated between biofluids, but we did identify numerous metabolites correlated within saliva and plasma, respectively. Pathway analysis revealed significant pathways enriched within plasma metabolites for several disease states. Our work provides a detailed analysis of the altered metabolome at various stages of liver disease while providing some context to altered pathways and relationships between metabolites.

## 1. Introduction

Liver cancer accounts for nearly 800,000 deaths annually with the number expected to rise in upcoming years [1]. More than 80% of primary liver cancers are diagnosed as hepatocellular carcinoma (HCC), with the minority being intrahepatic and hepatocellular cholangiocarcinomas [2]. Furthermore, nearly 80% of HCC cases occur against a background of cirrhosis, which can arise from multiple etiologies, but the majority are due to hepatitis B and C infections [3]. However, in recent years, non-alcoholic-associated fatty liver disease (NAFLD) has been increasing and is associated with HCC development. Nearly 25% of the general population are diagnosed with NAFLD, histologically defined as more than 5% hepatic steatosis [4]. NAFLD is associated with obesity and type 2 diabetes. In fact, 90% of individuals with obesity and without type 2 diabetes (BMI ≥ 30 kg/m^2^), and 65% with type 2 diabetes, are diagnosed with NAFLD [5,6]. In recent years, NAFLD has been redefined as metabolic dysfunction-associated fatty liver disease (MAFLD) to better reflect the underlying metabolic factors associated with its progression. NAFLD with simple steatosis can progress to nonalcoholic steatohepatitis (NASH) with chronic inflammation, predisposing the liver to cirrhosis and ultimately to the development of HCC [7]. Annual HCC incidence from NASH with cirrhosis is 1–2% [8]. Additionally, nearly 50% of NAFLD patients that develop HCC do not have evidence of cirrhosis, making screening a challenge for this population [9,10,11]. The current model for development and progression of NAFLD includes initial excess fat deposition in the liver [7]. This can ultimately result in lipotoxicity, oxidative stress, and mitochondrial dysfunction promoting an inflammatory environment in the liver that can progress to fibrosis and cirrhosis and/or HCC.

Development of biomarkers and new screening tools are crucial for the early diagnosis of these disease states, allowing for timely intervention to significantly slow or reverse disease progression. Recent evidence has suggested that fibrosis in NAFLD and NASH is reversible during primary stages through weight loss of >10% body weight [12]. In addition to weight loss, Resmetirom has been FDA-approved for treatment of NASH without cirrhosis [13]. Moreover, HCC can be more effectively treated in early stages when liver function is preserved, allowing for curative options such as liver resection, ablation, or transplantation. In fact, the five-year survival rate for early-stage HCC is more than 70%, but dips to <20% in the advanced stages of the disease [14,15,16]. Current guidelines recommend ultrasound of the liver typically with monitoring serum alpha fetoprotein levels for patients at-risk [17]. However, up to 40% of early-stage HCC cases are missed using these screenings [18]. In addition, HCC that develops against a background of NAFLD is generally identified at later stages than HCC with other etiologies, resulting in worse prognosis for this subset of patients [10,19]. Therefore, it is critical that accurate and non-invasive biomarkers can be made readily available to identify these patients.

Recent work has explored the use of metabolites for non-invasive biomarker discovery in biofluids such as saliva and plasma for various cancers. Here, we add to the growing field of the altered metabolome in liver diseases, including HCC [20,21]. We analyzed the saliva and plasma metabolome in healthy, NAFLD, cirrhotic, and HCC patients, and found significantly altered metabolites and metabolic pathways distinguishing liver disease states in both biofluids. Overall, we provide a detailed analysis of the altered metabolome across various liver disease pathologies.

## 2. Results

### 2.1. Saliva Metabolites Associated with Liver Disease

**Figure 1:**
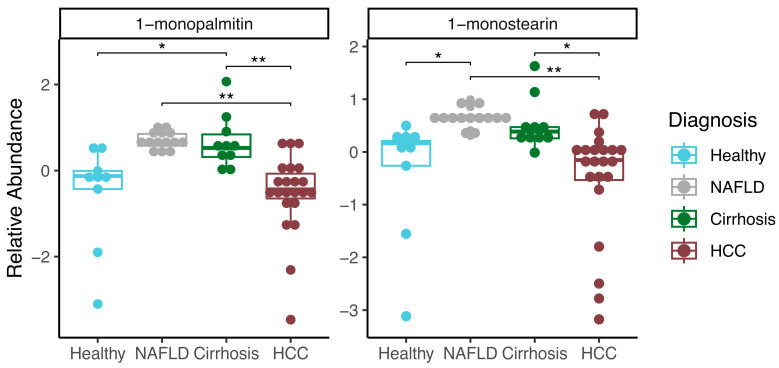
Salivary metabolomics from individuals with healthy, NALFD, cirrhosis, or HCC livers. Boxplots depicting the relative abundances of metabolites and significant associations among pairwise disease comparisons (* FDR *p* < 0.1, ** FDR *p* < 0.05).

We set out to identify minimally invasive biomarkers for liver diseases, which included NAFLD, cirrhosis, and HCC in different biofluids. The clinical characteristics of our patient cohorts are described in Table 1 and Table 2. A total of 111 salivary metabolites passed quality control (see Methods). We performed an ANOVA to identify significant associations among the four cohorts and each metabolite. This revealed 1-monopalmitin and 1-monostearin (FDR *p* < 0.05) as being significantly different between groups (Supplementary Table S2). Pairwise logistic regression revealed that 1-monopalmitin and 1-monostearin were significantly decreased in HCC compared to NAFLD (Log2FC: −1.578, −1.156) and cirrhosis (Log2FC: −1.391, −0.782), respectively (Figure 1, Supplementary Table S3). Additionally, 1-monostearin was significantly increased in NAFLD compared to in individuals in the healthy group (Log2FC: 0.699), while 1-monopalmitin was significantly increased in those with cirrhosis compared to those in the healthy group (Log2FC: 0.942). Neither metabolite was significantly associated with age, BMI, sex, or smoking status after adjusting for disease status (*p* > 0.1).

### 2.2. Plasma Metabolites Associated with Liver Disease

**Figure 2:**
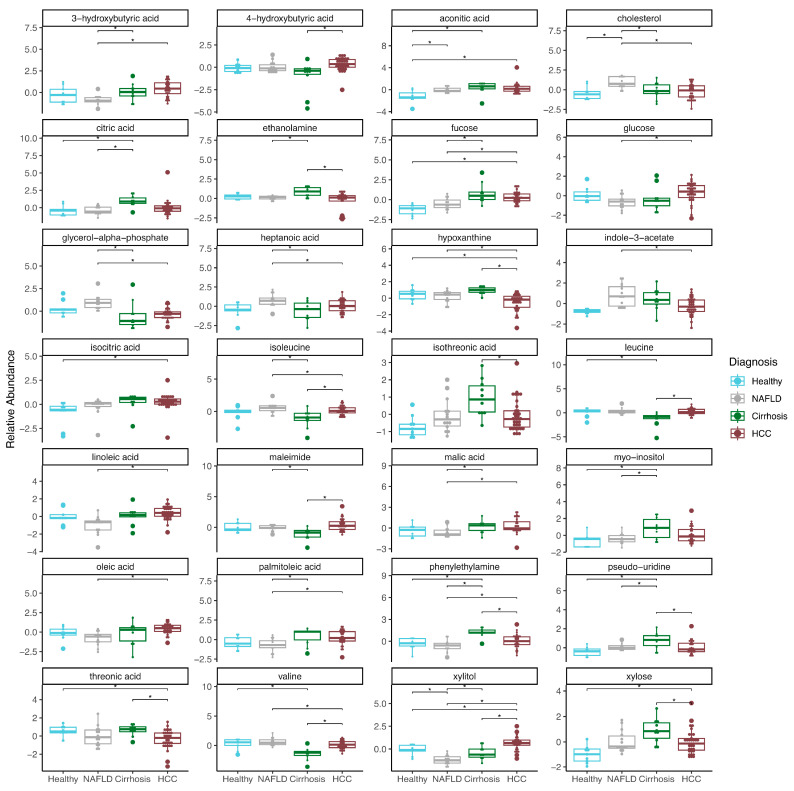
Plasma metabolomics from individuals with healthy, NALFD, cirrhosis, or HCC livers. Boxplots depicting the relative abundances of metabolites and significant associations among pairwise disease comparisons (* FDR *p* < 0.1).

We quantified 110 metabolites that passed QC in plasma, and 29 metabolites were associated with disease status (ANOVA: FDR *p* < 0.05) (Supplementary Table S4). Pairwise logistic regression of these metabolites revealed significant associations with many diagnosis comparisons. Twenty-six of the metabolites were significantly associated with HCC when compared to another diagnosis (Figure 2, Supplementary Table S5), and 22 of the metabolites were significantly associated with NAFLD when compared to another diagnosis.

Hypoxanthine and xylitol were the only metabolites significantly lower and higher, respectively, in HCC compared to all other diagnoses (Log2FC Healthy: −1.021, 1.106; NAFLD: −0.878, 2.74; Cirrhosis −1.684, 1.829) (FDR *p* < 0.05). In cirrhosis, phenylethylamine and pseudouridine were significantly increased compared to all other diagnoses, respectively (Log2FC Healthy: 2.096, 1.725; NAFLD: 2.525, 1.192; HCC: 1.669, 1.432). Of note, the relative abundance of cholesterol was significantly increased in NAFLD compared to Healthy (Log2FC: 1.918), Cirrhosis (Log2FC: 1.325), and HCC (Log2FC: 1.198) in plasma samples. Furthermore, the relative abundance of aconitic acid was significantly lower in the healthy group compared to any other liver disease (Log2FC NAFLD: −1.7; Cirrhosis: −2.912; HCC: −2.275). Of the 29 metabolites, isothreonic acid was significantly associated with age, while isoleucine, leucine and valine were associated with sex (FDR *p* < 0.1).

### 2.3. Metabolite Correlations in Saliva and Plasma

**Figure 3:**
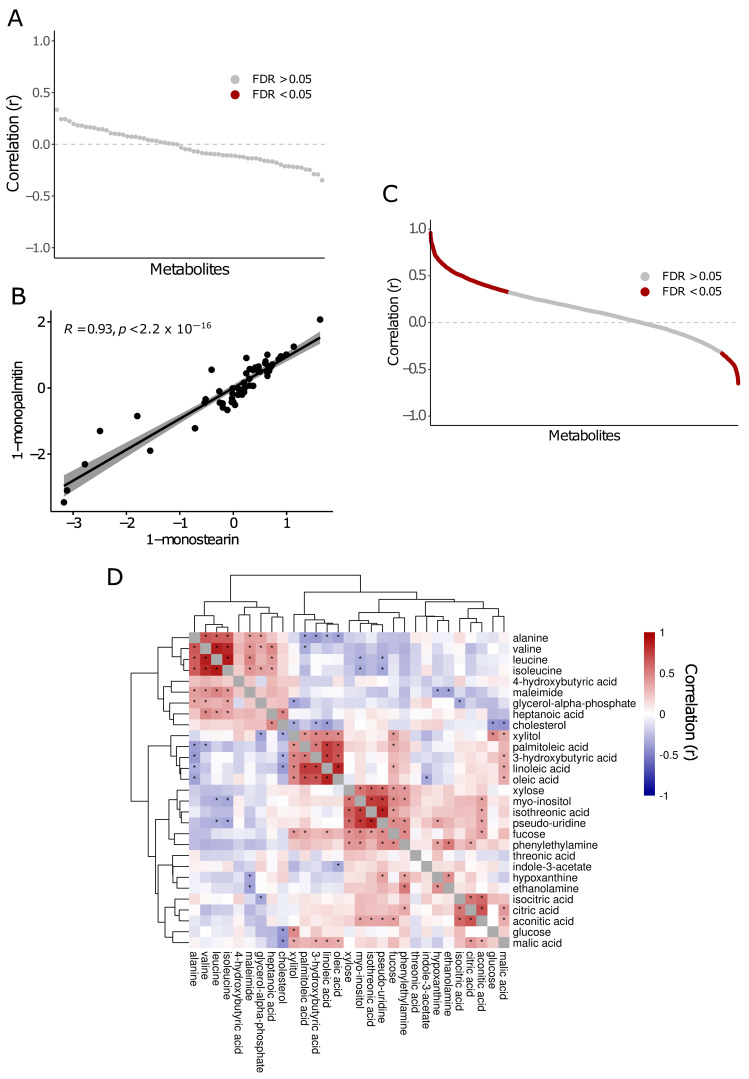
Metabolite correlations between and within saliva and plasma biofluids. (**A**) Scatterplot depicting correlations of relative metabolite abundances between plasma and saliva samples. No metabolites met the significance threshold of FDR *p* < 0.05 (red). (**B**) Correlation of 1-monopalmitin and 1-monostearin relative abundances within saliva samples. (**C**) Scatterplot depicting correlations of relative metabolite abundances within plasma samples. Significant correlations (FDR *p* < 0.05) are depicted in red. (**D**) Heatmap depicting clustered relative metabolite abundances within plasma samples. Heatmap colored by correlation (r). Asterisks depict significant metabolite correlations with an FDR *p* < 0.05.

Our cohort had matching plasma and saliva samples for 40 patients. Of the 110 and 111 metabolites measured in plasma and saliva, respectively, 65 were quantified in both. However, we did not observe any significant correlations between metabolites in the two biofluids, suggesting that each provides largely independent information (Figure 3A).

We also performed correlations among metabolites that were significant (ANOVA FDR *p* < 0.05) in saliva and plasma separately. In saliva, we found 1-monopalmitin and 1-monostearin abundances were significantly correlated (r = 0.93, *p*-value < 2.2 × 10^—16^), suggesting a potentially shared mechanism of regulation of these metabolites in saliva (Figure 3B).

Of the 29 significant metabolites in plasma (ANOVA FDR *p* < 0.05), we found 82/406 metabolite combinations to be significantly correlated (FDR *p* < 0.05) (Figure 3C) (Supplementary Table S6). This included 63 positive and 19 negative correlations. We clustered the correlation coefficients to identify similarly regulated metabolites (Figure 3D). Of note, all the branched chain amino acids (BCAAs), which include valine, leucine, and isoleucine were positively correlated (leucine-isoleucine: r = 0.88; leucine-valine: r = 0.86; isoleucine-valine: r = 0.82). Furthermore, we identified a positively correlated cluster which included xylitol, palmitoleic acid, linoleic acid, oleic acid, and 3-hydroxybutyric acid, as well as another cluster which included xylose, myo-inositol, isothreonic acid, pseudouridine, fucose, and phenylethylamine.

### 2.4. Enriched Metabolic Pathways Associated with Liver Disease

To gain a better understanding of the biological implications of the significantly altered metabolic abundances, we performed pathway analysis using MetaboAnalyst to identify pathways enriched in each type of liver disease [22]. After performing a logistic regression for each metabolite and disease status for each biofluid, metabolites with an unadjusted *p* < 0.05 were used as an input for pathway enrichment analysis (Supplementary Table S7).

**Figure 4:**
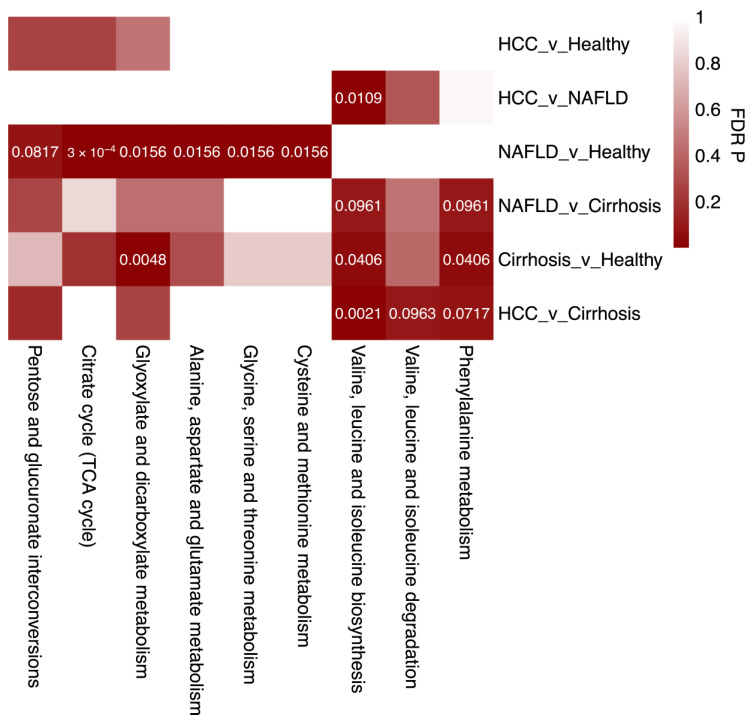
Over-represented pathways in liver diseases from plasma metabolomics. Heatmap depicting FDR of KEGG pathways over-represented in liver disease comparisons. Significant pathways (FDR *p* < 0.1) are depicted by numerical FDR values.

Significant salivary metabolites were not found to be enriched (FDR *p* < 0.1) in any pathway, whereas nine pathways were enriched with significant plasma metabolites (Figure 4). In NAFLD vs. Healthy, the following pathways were enriched: ‘The Citric Acid Cycle’ (FDR *p* = 0.0003), ‘glyoxate and dicarboxylate metabolism’ (FDR *p* = 0.0156), ‘alanine aspartate and glutamate metabolism’ (FDR *p* = 0.0156), ‘pentose and glucoronate interconversions’ (FDR *p* = 0.0817), ‘glycine, serin, and threonine metabolism’ (FDR *p* = 0.0156), and ‘cysteine and methionine metabolism’ (FDR *p* = 0.0156) pathways. The ‘valine, leucine, and isoleucine biosynthesis pathway’ was enriched in all comparisons besides HCC vs. Healthy, and NAFLD vs. Healthy. Interestingly, this same pathway, as well as the ‘valine, leucine, and isoleucine degradation pathway’, was enriched in HCC vs. Cirrhosis. Finally, the ‘phenylalanine metabolism pathway’ was enriched in all cirrhosis comparisons.

## 3. Discussion

The spectrum of chronic liver diseases continues to be a global health burden with a rise in incidence and mortality. Multiple etiologies contribute to the development of liver disease. Globally, HBV and HCV dominate this landscape, significantly contributing to cirrhosis, liver failure, and/or HCC. However, in recent years with the increase in obesity and type 2 diabetes, NAFLD has become more prevalent, with nearly 25% of the population diagnosed with this disease. Early stages of NAFLD with hepatic steatosis can impair liver function and, if left unchecked, can progress to a more severe form termed NASH with chronic inflammation and fibrosis, which can ultimately develop into cirrhosis and HCC.

The various etiologies of liver disease can progress in different ways via a combination of common and unique molecular mechanisms. For example, NAFLD and HBV infections can progress to HCC without presentation of cirrhosis, making screening challenging for different at-risk populations and necessitates the discovery of new biomarkers for individual liver disease etiologies to allow for timely intervention. In this study, we performed untargeted metabolomics of saliva and plasma to discover novel non-invasive biomarkers for NAFLD, cirrhosis, and HCC patients.

### 3.1. Salivary Metabolites Associated with Liver Disease

In saliva, we identified two metabolites, 1-monopalmatin and 1-monstearin, that were significantly lower in HCC when compared to cirrhosis and NAFLD. Moreover, we observed a positive correlation between these two monoglycerides in our saliva samples, possibly suggesting a similar mechanism of regulation in saliva. These two monoglycerides have previously been identified as biomarkers in serum for diabetes and obesity [23,24]. Furthermore, prior work has utilized these metabolites in a saliva model to help distinguish healthy, cirrhosis, and HCC patients [21]. More work is necessary to understand the role these monoglycerides play in liver disease.

### 3.2. Plasma Metabolites Associated with Liver Disease

Metabolomics in plasma revealed a wide array of significantly altered metabolites among the liver disease groups. In fact, we identified at least one metabolite for healthy, cirrhosis, NAFLD, and HCC that was significantly different when compared to all other diagnoses.

We identified two metabolites, cholesterol and xylitol, whose metabolic abundances were significantly different in NAFLD compared to all other diagnoses. Cholesterol is known to be associated with obesity. However, BMI did not significantly differ between groups in our cohort, and cholesterol was not significantly associated with BMI within our samples after adjusting for disease status. Of note, the relative abundance of xylitol was significantly different among all disease states except healthy, compared to cirrhosis. Past work has found xylitol to be significantly increased in the blood of patients with HCC compared to chronic liver disease with HBV or HCV infection [25]. Furthermore, xylitol was found to be a potential urine biomarker in a cohort of liver cirrhosis and HCC patients compared to healthy controls [26]. In a separate study, xylitol was found to be significantly decreased in the urine of individuals who were obese (BMI > 95th percentile) or who were diagnosed with NAFLD compared to individuals with a lower BMI (BMI 25th–85th percentile) [27]. Interestingly, numerous other metabolites in plasma (3-hydroxybutyric acid, fucose, glycerol-alpha-phosphate, heptanoic acid, isoleucine, malic acid, palmitoleic acid, and phenylethylamine) distinguished NAFLD from late-stage liver disease (Cirrhosis, HCC).

In the plasma of patients with cirrhosis, we identified pseudouridine and phenylethylamine as metabolites whose metabolic abundances were significantly different from those of all other disease groups. Additionally, we found valine, leucine, and isoleucine to be significantly different in patients with cirrhosis when compared to at least two out of the other three groups. Past work has described a decrease in circulating branched chain amino acids (BCAA) (valine, leucine, and isoleucine) in patients diagnosed with liver cirrhosis [28,29]. In other work, a decrease in BCAAs was found to be associated with mortality in liver disease patients [30]. All three BCAAs were significantly reduced in our cirrhosis cohort compared to HCC or NAFLD. Of note, the BCAAs were positively correlated with one another in our plasma samples. Typically, in patients with cirrhosis, a decrease in BCAAs correlated with an increase in aromatic amino acids (AAA) (phenylalanine, tyrosine, and tryptophan) known as the Fishers’ Ratio, a ratio which is associated with hepatic encephalopathy [31]. All three AAAs were measured in our untargeted metabolomics but did not pass QC (see Methods) for analysis. Interestingly, we found the valine, leucine, and isoleucine biosynthesis KEGG pathway to be over-represented in all cirrhosis disease comparisons (Cirrhosis vs. Healthy, NAFLD vs. Cirrhosis, HCC, vs. Cirrhosis), while the degradation pathway of these metabolites was enriched in the HCC vs. Cirrhosis comparison.

In this study, we found pseudouridine to be significantly increased in the plasma of our liver cirrhosis patients compared to healthy, NAFLD, and HCC patients. Pseudouridine is one of the most abundant RNA modifications which is carried out by a family of pseudouridine synthetases (PUS). Recent work has found PUS1 to be upregulated in various cancers including HCC, and may contribute to oncogenesis in HCC [32,33]. Hu et al. found overexpression of PUS1 to increase cell proliferation and tumor formation in cell lines and mouse models, respectively [34]. In their work, they proposed a mechanism of oncogenesis whereby oncogenic mRNAs are pseudouridinylated by PUS1, resulting in increased translation. In other work, pseudouridine has been found to be increased in the serum or urine of patients with HCC and in the serum of patients with liver cirrhosis likely to develop acute kidney injury [35,36,37].

We identified two metabolites, hypoxanthine and xylitol, whose metabolic abundances were significantly different in HCC when compared to healthy individuals or those with liver disease. Hypoxanthine is a metabolic intermediate in the purine catabolism pathway mediated by xanthine oxidoreductase (XDH). XDH is highly expressed in the liver but downregulated in HCC [37]. Low XDH levels are associated with poor prognosis in liver cancer and are believed to contribute to HCC progression [38,39,40]. Dysregulation of purine metabolism and decreased XDH activity is also associated with the progression of other cancers such as glioblastoma, lung, breast, ovarian, and colorectal cancers [40,41,42,43,44]. Past studies have found there to be elevated levels of hypoxanthine in the serum of HCC patients compared to cirrhosis among those with a viral hepatitis etiology [45,46]. However, other work has described serum hypoxanthine to be reduced in an HCC cohort compared to healthy participants [47]. Additionally, a decrease in hypoxanthine serum levels was observed in patients with liver cirrhosis when compared to healthy participants [45]. In urine, hypoxanthine was found to be reduced in HCC participants compared to a healthy control group [48]. The differences in hypoxanthine levels across studies may be attributed to cohort differences, including variations in HCC etiology. Ultimately, this underscores the necessity for further investigation into biomarkers for different HCC etiologies.

Addressing the complexities and progression of NAFLD, cirrhosis, and HCC requires a multifaceted approach to tackle early detection through the discovery of novel biomarkers. In this work, we identified multiple metabolites in plasma that are significantly associated with HCC, cirrhosis, and NAFLD, and provide some molecular insight into the downstream dysregulated pathways that may be affected. One limitation of our pilot study is the sample size of our cohorts. However, even with a limited number of samples, our analyses produced significant results due to large effect sizes, while limiting type I errors. Future studies with larger sample sizes will be needed to validate these findings. Ultimately, biomarker discovery in various stages and etiologies in the spectrum of liver disease will provide novel insights into mechanism, detection, and disease progression, leading to timely treatment and enhanced patient care.

## 4. Materials and Methods

### 4.1. Subject Recruitment

Saliva and blood samples were collected from a clinical cohort of 82 adult patients at the Cleveland Clinic between 2015 and 2022. Samples of each collected biofluid for saliva and plasma were as follows: Healthy (9,9), NAFLD (14,14), Cirrhosis (10,10), and HCC (22,34). Cirrhosis and HCC patients underwent liver transplantation or surgical resection for HCC at the Cleveland Clinic. Cirrhosis and HCC pathologies were confirmed via liver biopsy and histopathological assessment. Patients with NAFLD were recruited from the hepatology clinic at the Cleveland Clinic. Our healthy liver population came from liver transplant donors with no history of liver disease. The clinical characteristics of the study subjects can be found in Table 1 and Table 2. Hemoglobin, platelets, AST, ALT, ALP, bilirubin, albumin, PT-INR, glucose, and creatine described in Table 1 and Table 2 are routine values obtained from a chart review of the electronic medical records (EMR). Lab values were measured by the Cleveland Clinic Central Laboratory using standard clinical methodology. All participants provided informed written consent, the study conformed to the ethical guidelines of the 1975 Declaration of Helsinki, and it was approved by the Cleveland Clinic IRB (IRB #10-347).

### 4.2. Biofluid Collection and Mass Spectrometry

Blood and saliva samples were collected from subjects during a scheduled visit with their physician at the Cleveland Clinic. Saliva samples were collected from subjects using a DNA Genotek OMNIgene ORAL OM-610 (Ottawa, ON, Canada) saliva collection kit following a standard mouth rinse. Samples were subsequently frozen at −80 °C until processing. Blood samples were collected in EDTA tubes followed by centrifugation at 3000 rpm at 4 °C for 20 min to isolate plasma. Plasma samples were aliquoted in cryovials and stored at −80 °C until processing. Untargeted gas chromatography time of flight mass spectrometry (GC-TOF MS) was performed on the biofluids at West Coast Metabolomics (Davis, CA, USA) previously described in [20,21]. In brief, the analysis was performed using a Leco Pegasus IV mass spectrometer (St. Joseph, MI, USA, LECO Corporation) with unit mass resolution, operating at 17 spectra per second, scanning from 80 to 500 Da with an ionization energy of −70 eV and a detector voltage of 1800 V. The transfer line was maintained at 230 °C, while the ion source was set at 250 °C. The analytical GC column was protected by a 10-m empty guard column, which was trimmed in 20 cm increments whenever quality control (QC) samples of the reference mixture indicated contamination. This chromatography method was optimized for high-quality retention and separation of primary metabolite classes, including amino acids, hydroxyl acids, carbohydrates, sugar acids, sterols, aromatics, nucleosides, and amines. An automatic liner exchange was performed after every 10 injections to minimize sample carryover, particularly for highly lipophilic compounds like free fatty acids. Metabolite relative abundances were calculated using ChromaTOF software version 2.32 and the BinBase algorithm (rtx5).

### 4.3. Metabolomics Data Processing and QC

A total of 219 annotated metabolite abundances were measured for saliva and plasma samples. Metabolites with <30% high quality spectra in a single biofluid were removed from analysis (Supplementary Table S1). This left 110 metabolites for plasma and 111 metabolites for saliva. For metabolites without high quality spectra, the quantification mass at the specified retention index following subtraction of the local minimum noise was used. The relative abundances of metabolites in both biofluids exhibited a right skew (Supplementary Figures S1A and S2A). Therefore, they were log-transformed, scaled and, centered across each biofluid cohort to produce normal distributions (Supplementary Figures S1B and S2B). Due to the disparities in age, sex, BMI, and smoking status between groups, we aimed to ensure these factors did not influence differences in metabolite abundance. Therefore, linear regressions were performed to test for associations between each metabolite and age, BMI, sex, or smoking status while adjusting for disease status. *p*-values were adjusted for multiple hypothesis testing using a false discovery approach (FDR) and those with an FDR *p* < 0.1 were considered significant [49]. All statistical analyses were performed using the software R v.4.3.1 [50].

### 4.4. Metabolite Associations with Disease Status

We first performed an ANOVA on each metabolite and disease status (Healthy, Cirrhosis, NAFLD, HCC). *p*-values were adjusted for multiple hypothesis testing using an FDR approach [49]. An FDR *p* of <0.05 was considered significant. Metabolites significantly associated with disease status were tested pairwise using logistic regressions to identify significant associations of each metabolite and disease group. An FDR *p* of <0.1 was considered significant.

### 4.5. Metabolite Correlations in Biofluids

Forty patients in our cohort had matched plasma and saliva samples. Pearson’s Correlation was used to test for the correlation of the 65 metabolites measured in both saliva and plasma biofluids.

For metabolite correlations in individual biofluids, we performed a Pearson’s Correlation. When more than one test was performed, in the case of plasma metabolites, we adjusted the *p*-values for multiple hypothesis testing using an FDR approach, as described above.

### 4.6. Metabolite Pathway Analysis

Pairwise logistic regressions were performed to test for associations with each combination of disease groups and all metabolites that passed QC (110 for plasma and 111 for saliva). Metabolites that had an unadjusted *p* < 0.05 were used as the input for pathway enrichment analyses using MetaboAnalyst [22]. We tested for over-representation of metabolites in pathways contained in the Kyoto Encyclopedia of Genes and Genomes (KEGG) using the MetaboAnalyst webtool. FDR *p* < 0.1 were considered significantly enriched.

## 5. Conclusions

In summary, this pilot study focused on identifying novel, non-invasive metabolite biomarkers for NAFLD, cirrhosis, and HCC within plasma and saliva. We identified two salivary and 28 plasma metabolites that were significantly associated with at least one disease state. In plasma, we identified at least one metabolite in NAFLD, cirrhosis, and HCC that exhibited significant differences compared to other diagnoses, suggesting their potential as markers for individual disease states. Although no pathways were enriched in saliva comparisons, we identified nine pathways enriched for significant plasma metabolites.

We expanded our analyses to identify significant correlations of metabolites between, and within, plasma and saliva. We did not observe significant correlations between metabolites in the two biofluids, suggesting each offer largely unique information. However, we did observe significant correlations among metabolites within each separate biofluid suggesting a potentially shared mechanism of regulation of these metabolites. Overall, our work provides a detailed analysis of the altered metabolome at various stages of liver disease while providing some context to altered pathways and relationships between metabolites.

## 6. Patents

U.S. Provisional Patent Application No. 63/186,47. Salivary Metabolites Are Non-Invasive Biomarkers of HCC. Rotroff, D.M., Aucejo, F., and Hershberger, C. 2021.

## Figures and Tables

**Table 1:** Summary statistics for saliva study cohort. Alanine aminotransferase (ALT), aspartate aminotransferase (AST), alkaline phosphatase (ALP), prothrombin time international normalized ratio (PT-INR).

	Saliva			
Characteristic	Healthy, N = 9 ^1^	NAFLD, N = 14 ^1^	Cirrhosis, N = 10 ^1^	HCC, N = 22 ^1^
Age	34 (30, 44)	55 (42, 62)	64 (62, 66)	67 (61, 77)
Sex				
Male	5 (56%)	4 (29%)	7 (70%)	17 (77%)
Female	4 (44%)	10 (71%)	3 (30%)	5 (23%)
BMI	32.6 (28.4, 34.9)	30.9 (28.9, 35.1)	28.5 (27.7, 34.9)	28.2 (26.5, 32.3)
Cirrhosis Status				
Compensated	-	-	2 (20%)	7 (32%)
Mild	-	-	-	-
Decompensated	-	-	8 (80%)	7 (32%)
Unspecified	-	-	-	1 (4.5%)
None	9 (100%)	14 (100%)	-	7 (32%)
Cirrhosis Etiology				
Alcohol	-	-	5 (50%)	1 (4.5%)
Alcohol/HBV/HCV	-	-	-	1 (4.5%)
HBV/HCV	-	-	-	8 (36%)
NAFLD/NASH	-	-	5 (50%)	2 (9.1%)
Other	-	-	-	-
Origin Unknown	-	-	-	3 (14%)
None	9 (100%)	14 (100%)	-	7 (32%)
Child Pugh				
A	9 (100%)	14 (100%)	3 (30%)	18 (82%)
B	-	-	5 (50%)	4 (18%)
C	-	-	2 (20%)	-
Tumor Lesion				
Multiple	-	-	-	6 (27%)
Single	-	-	-	16 (73%)
Tumor Size				
<4	-	-	-	12 (55%)
≥4	-	-	-	10 (45%)
Smoker	2 (22%)	2 (14%)	7 (70%)	13 (59%)
Diabetes				
Type 2 DM	-	7 (50%)	5 (50%)	10 (45%)
Prediabetes	-	2 (14%)	-	-
No	9 (100%)	5 (36%)	5 (50%)	12 (55%)
Hypertension	1 (11%)	11 (79%)	8 (80%)	21 (95%)
Coronary Artery Disease	1 (11%)	4 (29%)	4 (40%)	5 (23%)
Hyperlipidemia	2 (22%)	10 (71%)	7 (70%)	9 (41%)
Psychiatric Disorder	1 (11%)	4 (29%)	5 (50%)	4 (18%)
COPD/Asthma/OSA	-	6 (43%)	4 (40%)	7 (32%)
Other Cancer	-	7 (50%)	1 (10%)	6 (27%)
Thyroid disease	-	3 (21%)	2 (20%)	4 (18%)
Other PMH	3 (33%)	14 (100%)	10 (100%)	19 (86%)
Ascites	-	1 (7.1%)	7 (70%)	7 (32%)
Encephalopathy	-	-	7 (70%)	6 (27%)
Mean Hemoglobin (g/dL)	14.50 (13.50, 14.80)	12.95 (12.48, 13.98)	10.95 (8.55, 13.95)	11.80 (10.23, 13.18)
Mean Platelets (k/uL)	250 (231, 289)	293 (253, 332)	99 (84, 118)	187 (143, 218)
Mean AST (U/L)	20 (18, 25)	36 (26, 46)	62 (41, 125)	119 (84, 232)
Mean ALT (U/L)	24 (16, 29)	46 (29, 53)	27 (24, 134)	125 (68, 291)
Mean ALP (U/L)	63 (54, 72)	97 (82, 195)	124 (79, 179)	114 (77, 144)
Mean Bilirubin, Total (mg/dL)	0.40 (0.30, 0.50)	0.50 (0.43, 0.58)	1.30 (1.10, 1.40)	1.00 (0.63, 1.48)
Mean Albumin (g/dL)	4.80 (4.30, 4.80)	4.40 (4.15, 4.50)	3.55 (3.03, 3.70)	3.50 (3.25, 3.78)
Mean PT-INR	1.00 (1.00, 1.10)	1.00 (1.00, 1.00)	1.20 (1.13, 1.20)	1.20 (1.10, 1.30)
Mean Glucose (mg/dL)	89 (87, 91)	97 (88, 110)	138 (122, 164)	122 (112, 140)
Mean Creatinine (mg/dL)	0.82 (0.81, 0.90)	0.82 (0.73, 0.93)	1.36 (0.71, 1.86)	0.92 (0.77, 1.29)

^1^ Median (IQR); n (%).

**Table 2:** Summary Statistics for plasma study cohort. Alanine aminotransferase (ALT), aspartate aminotransferase (AST), alkaline phosphatase (ALP), prothrombin time international normalized ratio (PT-INR).

	Plasma			
Characteristic	Healthy, N = 9 ^1^	NAFLD, N = 14 ^1^	Cirrhosis, N = 10 ^1^	HCC, N = 34 ^1^
Age	41 (30, 46)	55 (42, 62)	64 (62, 66)	68 (63, 74)
Sex				
Male	4 (44%)	4 (29%)	7 (70%)	26 (76%)
Female	5 (56%)	10 (71%)	3 (30%)	8 (24%)
BMI	32.8 (30.2, 34.9)	30.9 (28.9, 35.1)	28.5 (27.7, 34.9)	29.7 (26.5, 32.4)
Cirrhosis Status				
Compensated	-	-	2 (20%)	8 (24%)
Mild	-	-	-	1 (2.9%)
Decompensated	-	-	8 (80%)	8 (24%)
Unspecified	-	-	-	1 (2.9%)
None	9 (100%)	14 (100%)	-	16 (47%)
Cirrhosis Etiology				
Alcohol	-	-	5 (50%)	2 (5.9%)
Alcohol/HBV/HCV	-	-	-	2 (5.9%)
HBV/HCV	-	-	-	7 (21%)
NAFLD/NASH	-	-	5 (50%)	2 (5.9%)
Other	-	-	-	2 (5.9%)
Origin Unknown	-	-	-	3 (8.8%)
None	9 (100%)	14 (100%)	-	16 (47%)
Child Pugh				
A	9 (100%)	14 (100%)	3 (30%)	27 (79%)
B	-	-	5 (50%)	7 (21%)
C	-	-	2 (20%)	-
Tumor Lesion				
Multiple	-	-	-	13 (38%)
Single	-	-	-	21 (62%)
Tumor Size				
<4	-	-	-	14 (41%)
≥4	-	-	-	20 (59%)
Smoker	2 (22%)	2 (14%)	7 (70%)	23 (68%)
Diabetes				
Type 2 DM	-	7 (50%)	5 (50%)	16 (47%)
Prediabetes	-	2 (14%)	-	-
No	9 (100%)	5 (36%)	5 (50%)	18 (53%)
Hypertension	1 (11%)	11 (79%)	8 (80%)	27 (79%)
Coronary Artery Disease	1 (11%)	4 (29%)	4 (40%)	7 (21%)
Hyperlipidemia	2 (22%)	10 (71%)	7 (70%)	12 (35%)
Psychiatric Disorder	2 (22%)	4 (29%)	5 (50%)	12 (35%)
COPD/Asthma/OSA	-	6 (43%)	4 (40%)	12 (35%)
Other Cancer	-	7 (50%)	1 (10%)	12 (35%)
Thyroid disease	-	3 (21%)	2 (20%)	3 (8.8%)
Other PMH	4 (44%)	14 (100%)	10 (100%)	33 (97%)
Ascites	-	1 (7.1%)	7 (70%)	10 (29%)
Encephalopathy	-	-	7 (70%)	10 (29%)
Mean Hemoglobin (g/dL)	13.90 (13.50, 14.60)	12.95 (12.48, 13.98)	10.95 (8.55, 13.95)	12.05 (10.73, 13.08)
Mean Platelets (k/uL)	269 (231, 301)	293 (253, 332)	99 (84, 118)	197 (172, 231)
Mean AST (U/L)	20 (18, 24)	36 (26, 46)	62 (41, 125)	135 (96, 209)
Mean ALT (U/L)	20 (16, 29)	46 (29, 53)	27 (24, 134)	154 (82, 202)
Mean ALP (U/L)	63 (54, 72)	97 (82, 195)	124 (79, 179)	100 (69, 130)
Mean Bilirubin, Total (mg/dL)	0.40 (0.30, 0.50)	0.50 (0.43, 0.58)	1.30 (1.10, 1.40)	0.80 (0.63, 1.20)
Mean Albumin (g/dL)	4.60 (4.20, 4.80)	4.40 (4.15, 4.50)	3.55 (3.03, 3.70)	3.60 (3.20, 3.80)
Mean PT-INR	1.00 (1.00, 1.10)	1.00 (1.00, 1.00)	1.20 (1.13, 1.20)	1.10 (1.10, 1.20)
Mean Glucose (mg/dL)	89 (88, 96)	97 (88, 110)	138 (122, 164)	123 (108, 140)
Mean Creatinine (mg/dL)	0.82 (0.69, 0.84)	0.82 (0.73, 0.93)	1.36 (0.71, 1.86)	0.94 (0.69, 1.10)

^1^ Median (IQR); n (%).

## Data Availability

The data presented in this paper are available in Supplementary Tables S8–S10.

## Supplementary Materials

The following supporting information can be downloaded at: https://www.mdpi.com/article/10.3390/ijms251810144/s1.
